# Effects of Rapamycin on Insulin Brain Endothelial Cell Binding and Blood–Brain Barrier Transport

**DOI:** 10.3390/medsci9030056

**Published:** 2021-08-25

**Authors:** Steven Nguyen, William A. Banks, Elizabeth M. Rhea

**Affiliations:** 1 University of Washington, Seattle, WA 98195, USA; steeven.nguyen1102@gmail.com; 2 Department of Medicine, Division of Gerontology and Geriatric Medicine, University of Washington, Seattle, WA 98195, USA; wabanks1@uw.edu; 3 Research and Development, Veterans Affairs Puget Sound Health Care System, Seattle, WA 98108, USA

**Keywords:** rapamycin, insulin, blood–brain barrier

## Abstract

Rapamycin is an exogenous compound that has been shown to improve cognition in Alzheimer’s disease mouse models and can regulate pathways downstream of the insulin receptor signaling pathway. Insulin is also known to improve cognition in rodent models of Alzheimer’s disease. Central nervous system (CNS) insulin must first cross the blood–brain barrier (BBB), a specialized network of brain endothelial cells. This transport process is regulated by physiological factors, such as insulin itself, triglycerides, cytokines, and starvation. Since rapamycin treatment can alter the metabolic state of rodents, increase the circulating triglycerides, and acts as a starvation mimetic, we hypothesized rapamycin could alter the rate of insulin transport across the BBB, providing a potential mechanism for the beneficial effects of rapamycin on cognition. Using young male and female CD-1 mice, we measured the effects of rapamycin on the basal levels of serum factors, insulin receptor signaling, vascular binding, and BBB pharmacokinetics. We found chronic rapamycin treatment was able to affect basal levels of circulating serum factors and endothelial cell insulin receptor signaling. In addition, while acute rapamycin treatment did affect insulin binding at the BBB, overall transport was unaltered. Chronic rapamycin slowed insulin BBB transport non-significantly (*p* = 0.055). These results suggest that rapamycin may not directly impact the transport of insulin at the BBB but could be acting to alter insulin signaling within brain endothelial cells, which can affect downstream signaling.

## 1. Introduction

Rapamycin, also known as sirolimus, is a compound that has been extensively studied for its impact on life span and health span across multiple species [1]. It primarily acts as an inhibitor of the ubiquitous signaling protein mammalian target of rapamycin (mTOR), which regulates growth and metabolism. There are many studies that have investigated the beneficial effect of rapamycin on life span extension, particularly in relation to cognitive decline and Alzheimer’s disease. These studies, summarized by Kaeberlein and Galvan [2], show rapamycin treatment in various mouse models of Alzheimer’s disease can reduce amyloid-β deposition, pathogenic tau phosphorylation, and neurofibrillary tangles; restore cerebral blood flow; preserve the blood–brain barrier’s (BBB) integrity; and improve cognitive function.

Increasing central nervous system (CNS) insulin improves cognitive function as well. Brain insulin levels are dependent on BBB insulin transport. Therefore, if the basal BBB transport of insulin can be increased, CNS levels can be increased, leading to improvements in memory. Indeed, there are many intersections between metabolism, insulin signaling, and rapamycin [3]. Rapamycin acts as a starvation mimetic, triggering a physiological unfed state. Starvation in mice increases insulin BBB transport [4]. Rapamycin also increases plasma triglyceride levels following a 14-day sirolimus treatment in humans [5] and in mice following longer rapamycin treatment [6]. Plasma triglycerides are known to increase insulin BBB transport in mice [4]. Cytokines can also increase insulin BBB transport [7]. Additionally, changes in interactions of insulin at the BBB can indirectly impact signaling within the CNS. That is, the binding of insulin to the insulin receptor located on endothelial cells can affect the interactions of brain endothelial cells with other signaling factors by affecting cell adhesion molecules [8]. Therefore, we hypothesized rapamycin would increase the amount of insulin BBB transport or affect the BBB interactions, which could be a mechanism for improved cognition. We randomly divided young male and female mice to receive either a vehicle or rapamycin daily injections for two weeks prior to the measurement of insulin signaling, vascular binding, and BBB pharmacokinetics. We also included a group of mice that received a single, acute injection of rapamycin to determine if there were any acute effects on insulin BBB transport.

## 2. Materials and Methods

### 2.1. Animals

Male and female CD-1 mice (7 weeks old) were purchased from Charles River Laboratories (Seattle, WA, USA). For chronic treatments, rapamycin intraperitoneal (IP) injections began at 8 weeks of age daily for 2 weeks. The mice were studied at 10 weeks of age. The mice had free access to water and food and were kept on a 12/12 h light/dark cycle. The mice were housed in groups of 4 mice per cage. Body weights were measured every two to three days. The local Institutional Animal Care and Use Committee (IACUC) at the Veteran Affairs Puget Sound approved all experimental animal procedures performed in this study. The study was performed at a facility approved by the Association for Assessment and Accreditation of Laboratory Animal Care International (AAALAC).

### 2.2. Chronic Rapamycin Injection Treatment

Rapamycin (LC Laboratories, Woburn, MA, USA) was prepared similar to the previously published studies [9]. Briefly, rapamycin was dissolved in dimethyl sulfoxide (DMSO, Sigma-Aldrich, St. Louis, MO, USA) to a stock concentration of 100 mg/ml. For the injectate, the 100 mg/mL rapamycin solution was diluted in a 5% Tween 80 (Sigma-Aldrich), 5% poly(ethylene glycol) 400 (PEG 400, Sigma-Aldrich) solution to create a final 1.2 mg/mL concentration. The vehicle was prepared by creating a 1.2% DMSO solution in 5% Tween 80, 5% PEG 400. The mice were randomly divided into two groups and received either a daily vehicle or rapamycin (8 mg/kg) IP injections for 2 weeks. The dosing was chosen based on what is currently used throughout the literature [9] as well as reports regarding similarities on the average trough levels of rapamycin between mice and humans [10].

### 2.3. Radioactive Labeling

Ten micrograms of human insulin (Sigma-Aldrich) were diluted in 40 µL 0.25 M chloride-free sodium phosphate buffer (PB) and labeled with 1 mCi ^125^I (PerkinElmer, Waltham, MA, USA) through the chloramine-T (Sigma-Aldrich) method. The reaction started with the addition of 10 μg chloramine T in 0.25 M PB and terminated 1 min later with the addition of 100 µg sodium metabisulfite. Albumin (1 mg, Sigma-Aldrich) was labeled with 1 mCi ^99m^Tc (GE Healthcare, Seattle, WA, USA) after combination with 120 µg stannous tartrate and 20 μL 1M HCl in 500 µL deionized water for a 20 min reaction. Both ^125^I-insulin and ^99m^Tc-albumin were purified via a column of Sephadex G-10 beads (Sigma-Aldrich) in 0.25 M PB. The protein labeling was characterized by 15% trichloroacetic acid (TCA) precipitation. Greater than 90% radioactivity in the precipitated fractions was consistently observed for insulin and albumin. 

### 2.4. Intravenous Injection Study

Following 2 weeks of daily the vehicle or rapamycin IP injections, the mice were anesthetized with 0.15 mL of 40% urethane and the right jugular vein and left carotid artery were exposed. The blood glucose was measured via the tail vein (AlphaTrak2, Abbott Laboratories, Chicago, IL, USA). The mice received an intravenous (i.v.) injection of a 0.2 mL solution containing 1 × 10^6^ cpm of ^125^I-insulin and 5 × 10^5^ cpm of ^99m^Tc-albumin in 1% bovine serum albumin in lactated Ringer’s (BSA-LR) directly into the jugular vein. The radioactivity circulated for times between 1–10 min, after which the carotid artery was cut to collect blood. The mice were immediately decapitated and the whole brain was removed and weighed. The blood was centrifuged at 5400 g for 10 min and the amount of radioactivity in the serum (50 µL) and the whole brain was counted in a gamma counter (Wizard2, PerkinElmer, Waltham, MA, USA). Two separate cohorts of mice were combined in the final analyses. In a separate cohort of mice, the effect of a single, acute rapamycin injection was studied. Naïve male mice were anesthetized as described. The vehicle or rapamycin (400 ng) was injected intravenously and allowed to circulate for 10 min. This dose was chosen based to approximately match a previous study reporting blood rapamycin levels following an 8 mg/kg body weight IP injection (100 ng/mL) [9]. Following the 10 min circulation period, the ^125^I-insulin and ^99m^Tc-albumin solution was injected as described above.

### 2.5. Transcardiac Brain Perfusion

Following the rapamycin treatment for 2 weeks, the thoracic cavity was opened, the heart was exposed, both jugulars were severed, and the descending thoracic aorta was clamped. A 26-gauge butterfly needle was inserted into the left ventricle of the heart and a freshly prepared Zlokovic’s buffer (7.19 g/L NaCl, 0.3 g/L KCl, 0.28 g/L CaCl_2_, 2.1 g/L NaHCO_3_, 0.16 g/L KH_2_PO_4_, 0.17 g/L anhydrous MgCl_2_, 0.99 g/L D-glucose, and 1% BSA) containing 2 × 10^5^ cpm ^125^I-insulin was infused at a rate of 2 mL/min for 1–5 min. Perfusate was collected throughout the study to determine the average cpm/µL of perfusate. After perfusion, the whole brain was weighed and the amount of radioactivity was counted in a gamma counter (Wizard2, PerkinElmer). The brain/perfusate ratios were calculated by dividing the cpm in a gram of brain by the cpm in a µL of perfusate to yield the units of µL/g.

### 2.6. Multiple-Time Regression Analysis

A multiple-time regression analysis was used as detailed previously [11,12] to calculate the rate of unidirectional influx for ^125^I-insulin. For the i.v. study, the brain/serum (B/S) ratios for ^125^I-insulin are corrected for vascular space by subtracting the corresponding ratio for ^99m^Tc-albumin, yielding a delta B/S ratio. The exposure time is calculated by the formula:

(1) $$Exposure\;time=\frac{\int_{0}^{t}Cp\left(t\right)dt}{Cp\left(t\right)}$$

where Cp(t) is the level of radioactivity (cpm) in the serum at time (t). The exposure time corrects for the clearance of peptide from the blood. The B/S ratios are plotted against the exposure time to calculate the influx of ^125^I-insulin using the same formula described by Blasberg et al. [11]:

(2) $$\frac{Am}{Cpt}=Ki\;(\frac{\int_{0}^{t}Cp\left(t\right)dt}{Cp\left(t\right)})+Vi$$

where Am is the level of radioactivity (cpm) per g of brain tissue at time t, Cpt is the level of radioactivity (cpm) per μL serum at time t, K_i_ (μL/g-min) is the unidirectional solute influx from blood to brain, and V_i_ (μL/g) is the level of rapid and reversible binding for the brain vasculature. The slope of the linearity measures the K_i_ and is reported with its standard error term. The y-intercept of the linearity measures the V_i_, the initial volume of distribution in brain at t = 0 [11]. For cardiac perfusion studies, the latter formula is employed, using the brain/perfusate ratios for ^125^I-insulin and the clock time is used in place of the exposure time.

### 2.7. Biochemical Tissue Processing

A separate cohort of male and female mice (*n* = 4/group) were treated for 2 weeks with vehicle or 8 mg/kg body weight rapamycin as described above. Following a 6 h fast, blood glucose was taken. The mice were anesthetized as described above, the thoracic cavity was opened, and blood was collected from the descending aorta. The heart was exposed, both jugulars were severed, and the descending thoracic aorta was clamped. The brain vasculature was washed out by perfusing 20 mL of ice cold LR into the left ventricle of the heart. The brain was removed and split into the right and left hemispheres. The aorta was also removed. The blood was centrifuged at 5400 g for 10 min and the serum was collected. All the tissues were snap frozen in liquid nitrogen and stored at −80 °C until further processing.

The right hemisphere was crushed with a mortar and pestle in liquid nitrogen and approximately 100 mg of tissue was homogenized in radioimmunoprecipitation assay (RIPA) buffer (150 mM NaCl, 0.5% deoxycholic acid, 0.1% sodium dodecyl sulfate (SDS), 20 mM Tris HCl, and 2 mM ethylenediaminetetraacetic acid (EDTA)) plus freshly added 1/100 dilutions of a protease inhibitor cocktail (Sigma-Aldrich), 1mM sodium orthovanadate, and a phosphatase inhibitor (1 tablet PhosSTOP per 10 mL buffer) (Roche, Basel, Switzerland). The aortas were homogenized in the same lysis buffer. The samples were sonicated at 40% amplitude prior to centrifugation at 12,000× *g* for 10 min at 4 °C. The supernatants were collected and frozen at −80 °C. The protein was measured using the Pierce BCA Protein Assay Kit (Thermo Fisher Scientific, Rockford, IL, USA).

### 2.8. Measurement of Hormone Levels

A panel of 8 metabolic hormones was measured in the brain lysate and serum using a Bio-Plex Pro Mouse Diabetes Assay (Bio-Rad Laboratories, Hercules, CA, USA): ghrelin, glucose-dependent insulinotropic peptide (GIP), glucagon-like peptide-1 (GLP-1), glucagon, insulin, leptin, plasminogen activator inhibitor-1 (PAI-1), and resistin. The serum was diluted 1:4 with the kit diluent. Twenty µg/µL protein lysate samples were measured from the right hemisphere. The samples were measured according to the manufacturer and read on a Bio-Plex 200 (Bio-Rad Laboratories).

### 2.9. Western Immunoblotting

The protein lysates were solubilized and denatured in NuPAGE sample buffer (Invitrogen, Grand Island, NY, USA), warmed for 10 min at 70 °C, and resolved on a 4–12% Bis-Tris gel (Invitrogen). The protein was then transferred to nitrocellulose membranes using the iBlot transfer system (Invitrogen). The membranes were blocked with 5% BSA in 0.1% Tween-20 in tris-buffered saline (TBS-T) (BSA/TBS-T) for 1 h. The membranes were incubated in primary antibodies from Cell Signaling Technologies (Danvers, MA, USA) for modifications in insulin signaling (phosphorylated protein kinase B [pAkt Ser^473^], total protein kinase B [Akt], mitogen-activated protein kinase [MAPK], and β-actin) prepared in BSA/TBS-T overnight at 4 °C. The membranes were washed in TBS-T and then probed with the respective secondary antibodies conjugated to horseradish peroxidase (Jackson Labs, West Grove, PA, USA) for 1 h at room temperature. The membranes were washed with TBS-T and then illuminated with ECL Prime Western Blotting Detection Reagent (Amersham, GE Life Sciences, Piscataway, NJ, USA). Following the band visualization on the ImageQuant LAS4000 CCD imaging system (GE Life Sciences), the blots were stripped with Restore Western Blot Stripping Buffer (Thermo Scientific) and re-probed for the total protein levels or β-actin. A densitometric analysis of bands was performed using ImageQuant TL (IQTL) software (GE Life Sciences, Piscataway, NJ, USA). The band intensities of phosphorylated proteins were normalized to the band intensities of the corresponding total protein levels, and the ratios of phosphorylated/total protein were normalized to the male vehicle group.

### 2.10. Measurement of Serum Cytokines

A panel of 23 cytokines was measured in the brain protein lysate and serum using a mouse Bio-Plex Pro Mouse Cytokine Assay (Bio-Rad Laboratories): interleukin (IL)-1α; IL-1β; IL-2; IL-3; IL-4; IL-5; IL-6; IL-9; IL-10; IL-12(p40); IL-12(p70); IL-13; IL-17; eotaxin (CCL11); granulocyte colony-stimulating factor (G-CSF); granulocyte-macrophage colony-stimulating factor (GM-CSF); interferon (IFN)-γ; keratinocyte chemoattractant (KC) (CXCL1); monocyte chemoattractant protein (MCP)-1 (CCL2); macrophage inflammatory protein (MIP)-1α (CCL3); MIP-1β (CCL4); and regulated on activation, normal T cell expressed and secreted (RANTES; CCL5) and tumor necrosis factor (TNF)-α). The serum samples were diluted 1:4 with the kit sample diluent. The samples were measured according to the manufacturer and read on a Bio-Plex 200 (Bio-Rad Laboratories).

### 2.11. Statistics

A regression analysis and other statistical analyses were performed with the use of Prism 8.0 (GraphPad Software Inc., San Diego, CA, USA). Body weight means were reported with their standard error terms and compared by a mixed-effects analysis followed by Sidak’s post hoc test. All the other basic biochemical comparisons were performed using a two-way ANOVA followed by Sidak’s post hoc test to compare the effect of sex and rapamycin treatment. Two-way ANOVA analyses are listed in the manuscript text while post hoc differences are designated on the figures with symbols and in the figure legends. For the pharmacokinetic studies, the slope of the linear regression lines (K_i_), reported with their correlation coefficients (r), and y-intercepts (V_i_) were compared statistically with the Prism 8.0 software package, as described [13].

## 3. Results

### 3.1. Acute Rapamycin Injection

**Figure 1:**
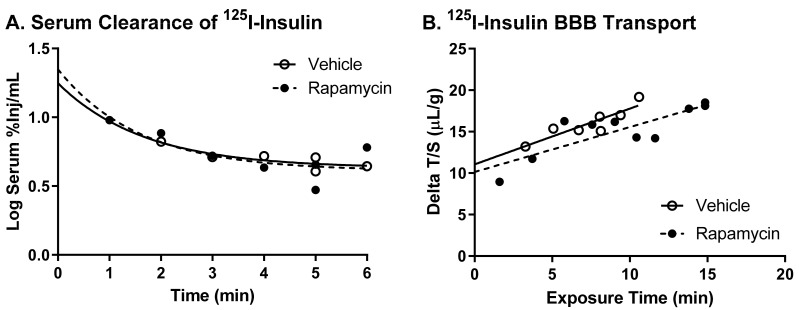
Effect of an acute single intravenous (i.v.) injection of rapamycin on insulin pharmacokinetics. (**A**) Serum clearance of ^125^I-insulin and (**B**) the transport rate of ^125^I-insulin across the blood–brain barrier (BBB) was unaffected by a 10 min i.v. pre-injection of rapamycin in male CD-1 mice.

We first determined if an acute increase in the blood rapamycin levels had an impact on the basal insulin BBB transport. We gave an i.v. injection of rapamycin to elevate the blood levels similar to those found after an IP injection [9], based on an approximate mouse blood volume of 4 mL. Ten minutes following an acute administration of 400 ng rapamycin directly into the circulation, there was no effect on the serum clearance for ^125^I-insulin (Figure 1A). In addition, the rate of insulin BBB transport (K_i_), 0.67 ± 0.2 µL/g-min for the vehicle and 0.54 ± 0.1 µL/g-min for rapamycin, was unchanged (Figure 1B, Table 1, *p* = 0.63). However, there was a decrease in the amount of ^125^I-insulin vascular binding (V_i_) with the rapamycin injection, 10.1 ± 1.3 µL/g, compared to the vehicle, 11.1 ± 1.6 µL/g vehicle (*p* = 0.02, Table 1), which is indicative of alterations in the vascular binding sites for ^125^I-insulin.

### 3.2. Effect of Chronic Rapamycin Treatment on Metabolic Factors

**Figure 2:**
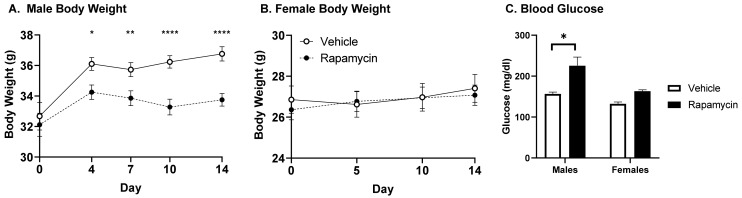
Metabolic effects of two weeks of rapamycin treatment. There is a significant effect of rapamycin on weight gain in (**A**) males only (*n* = 24–34) and not (**B**) females (*n* =18–19). (**C**) Blood glucose levels were taken from the mice following a 6 h fast. There was a significant increase in fasting blood glucose with the rapamycin treatment only in males (* *p* < 0.05); *n* = 12–16/group. The data represent combined data from 2–3 separate cohorts for each sex and is expressed as the mean ± SEM. * *p* < 0.05, ** *p* < 0.005, **** *p* < 0.0001

To investigate the transport of insulin across the BBB following chronic rapamycin IP injections, we treated mice daily with the vehicle or rapamycin for 2 weeks. Since rapamycin is known to have sex differences, we investigated the effect of rapamycin in males and females. We found 2 weeks of rapamycin treatment impacted body weight gain in males only (Figure 2A). In addition, we measured the fasting blood glucose levels following the rapamycin or vehicle treatment from two separate cohorts (Figure 2C). The males treated with rapamycin had significantly greater mean fasting blood glucose levels compared to the vehicle treated males (226 mg/dL vs. 157 mg/dL, *p* < 0.001). The females did not have the same increase following the rapamycin treatment (*p* = 0.19). 

**Figure 3:**
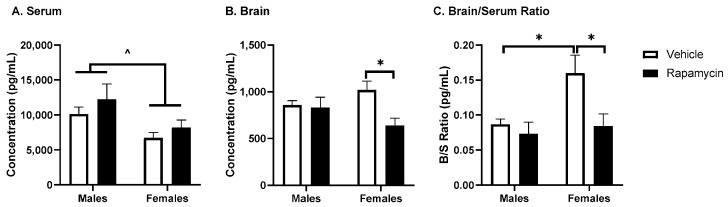
Insulin concentrations in serum and the brain following rapamycin treatment. Immunoactive insulin levels in serum and the whole brain were measured using the Bio-Plex Mouse Diabetes Panel Assay. The concentration in (**A**) serum and (**B**) the brain was analyzed to determine (**C**) the brain/serum ratio. The brain samples represent insulin levels from 1000 µg protein (whole brain lysate). Two-way ANOVA differences are indicated with ^ *p* < 0.05; post hoc analysis * *p* < 0.05; *n* = 3–4/group.

**Figure 4:**
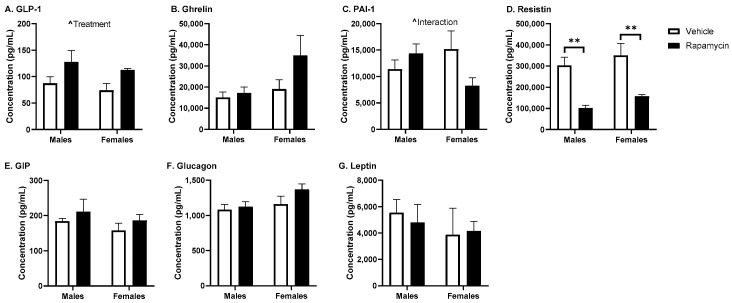
Serum metabolic hormone levels following rapamycin treatment. The serum was collected following a 6 h fast. (**A**) glucagon-like peptide-1 (GLP-1), (**B**) ghrelin, (**C**) plasminogen activator inhibitor-1 (PAI-1), (**D**) resistin, (**E**) glucose-dependent insulinotropic peptide (GIP), **F**) glucagon, and **G**) leptin were measured using the Bio-Rad mouse diabetes panel. Two-way ANOVA differences are indicated with ^ *p* < 0.05; post hoc analysis * *p* < 0.05; *n* = 4/group.

Based on these differences in body weight and blood glucose due to the rapamycin treatment between sexes, we decided to measure the serum levels of the metabolic hormones. The serum insulin levels were different due to sex (*p* = 0.012, F (1, 11) = 8.94, Figure 3A). In the brain, while there was a treatment effect of rapamycin (*p* = 0.034, F (1, 11) = 5.83, Figure 3B), the post hoc analysis revealed significant differences in females only (*p* = 0.014). This resulted in a significant effect of sex and the rapamycin treatment in the basal brain/serum insulin ratio in females compared to the vehicle (sex *p* = 0.046, F (1, 11) = 5.05; treatment *p* = 0.04, F (1, 11) = 5.71). The other serum metabolic hormones affected by treatment and/or sex included GLP-1 (treatment *p* = 0.015, F (1, 12) = 7.97, Figure 4A) and PAI-1 (interaction = 0.046, F (1, 12) = 4.83, Figure 4C). Rapamycin significantly decreased the serum resistin levels in both males and females (*p* < 0.0001, F (1, 12) = 31.36; post hoc *p* < 0.01, Figure 4D). There was no effect of sex or the rapamycin treatment on ghrelin, GIP, glucagon, or leptin levels. 

**Figure 5:**
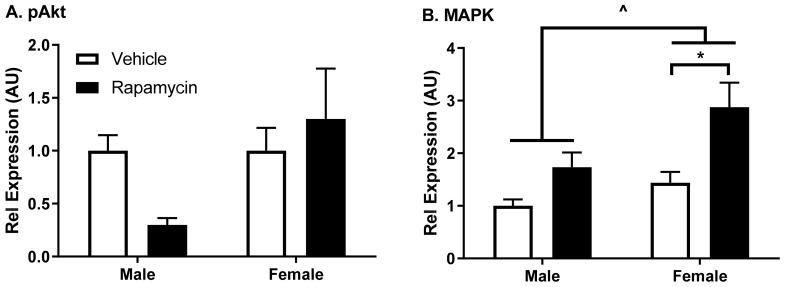
Insulin signaling protein levels in the aorta following rapamycin treatment. (**A**) phosphorylated protein kinase B (pAkt (Ser^473^)) levels relative to total protein kinase B (Akt) levels. (**B**) Mitogen-activated protein kinase (MAPK) levels were significantly increased due to sex (two-way ANOVA ^ *p* < 0.05) and due to the rapamycin treatment, particularly in females (post hoc analysis * *p* < 0.05); *n* = 3–4/group (Figure S1).

As the BBB is primarily comprised of endothelial cells, we wanted to investigate the impact of rapamycin treatment on endothelial cells. Therefore, we investigated insulin signaling protein changes in the aorta, a predominantly endothelial cell structure. We found a trend in decreased levels of phosphorylated Akt (pAkt Ser^473^) only in males (interaction *p* = 0.0918, F (1, 12) = 3.36, Figure 5A). We observed a significant increase in MAPK protein expression relative to β-actin in males and females due to sex (*p* = 0.031, F (1, 11) = 6.14) and with the rapamycin treatment (*p* = 0.006, F (1, 11) = 11.60, Figure 5B). 

### 3.3. Effect of Chronic Rapamycin Treatment on Serum Cytokine Expression

**Figure 6:**
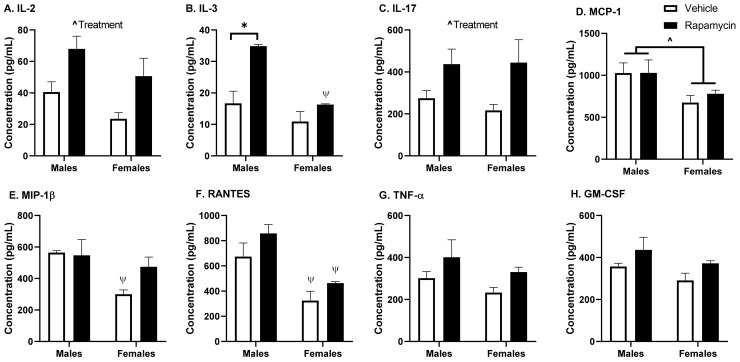
Serum cytokine levels following rapamycin treatment. The serum was collected following a 6 h fast. The rapamycin treatment significantly affected interleukin (IL) (**A**) IL-2, (**B**) IL-3, and (**C**) IL-17 levels. Sex significantly affected (**D**) monocyte chemoattractant protein (MCP)-1, (**E**) macrophage inflammatory protein (MIP)-1β, and (**F**) regulated on activation, normal T cell expressed and secreted (RANTES) levels. There was a trend (*p* < 0.07) towards an effect of treatment on (**G**) tumor necrosis factor (TNF-α) and (**H**) granulocyte-macrophage colony-stimulating factor (GM-CSF levels). Two-way ANOVA ^ *p* < 0.05; post hoc analysis * *p* < 0.05 as marked or ^Ψ^ *p* < 0.05 vs. sex within the treatment group; *n* = 3/group.

Inflammatory cytokines are also known to affect BBB transport, in particular insulin [7]. The serum cytokine expression is reported in Figure 6. The rapamycin treatment increased serum IL-2 levels (*p* = 0.009, F (1, 8) = 11.72, Figure 6A) and IL-17 (*p* = 0.023, F (1, 8) = 7.96, Figure 6C). IL-3 levels were increased significantly due to the rapamycin treatment (*p* = 0.004, F (1, 7) = 17.3, Figure 6B) and sex (*p* = 0.004, F (1, 7) = 18.4), with post hoc differences in males (*p* = 0.007) due to the treatment. MCP1, MIP-1β, and RANTES levels were affected by sex (*p* = 0.025, F (1, 8) = 7.54, Figure 6D; *p* = 0.024, F (1, 8) = 7.68, Figure 6E; *p* = 0.001, F (1, 8) = 24.6, Figure 6F, respectively). There were post hoc analysis differences in males versus females in the vehicle treated mice for MIP-1β (*p* = 0.030) and in males versus females for RANTES in both treatment groups (vehicle *p* = 0.02, rapamycin *p* = 0.01). There was a trend towards an increase in TNF-α, RANTES, and GM-CSF due to treatment, but these were not statistically significant (0.05 < *p* < 0.07). 

### 3.4. Impact of Chronic Rapamycin Treatment on Insulin BBB Transport

**Figure 7:**
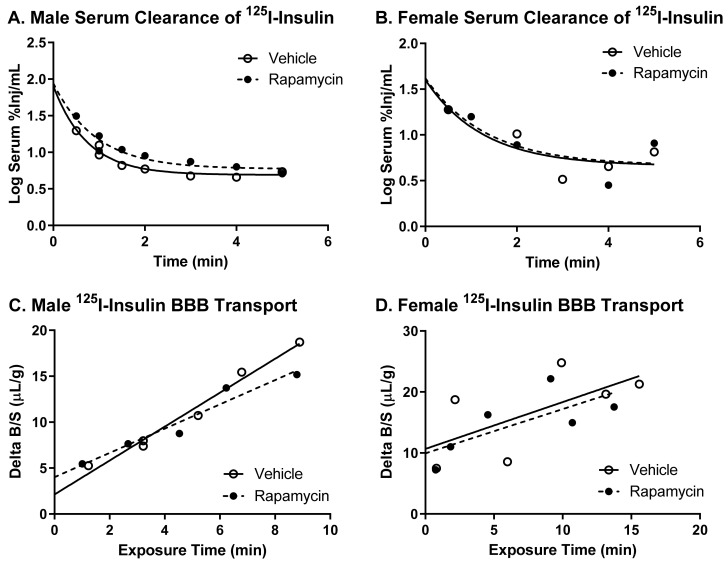
Effect of chronic rapamycin treatment on insulin pharmacokinetics. Rapamycin had no effect on ^125^I-insulin serum clearance in (**A**) males or (**B**) females. There were no statistical differences in ^125^I-insulin BBB transport either in (**C**) males or (**D**) females. Only the linear transport is shown. Pharmacokinetic data is represented in Table 2.

To investigate the effect of chronic rapamycin treatment on basal insulin pharmacokinetics, we measured the levels of ^125^I-insulin in the serum and the brain following the two-week treatment period. We investigated both males and females due to the metabolic differences between the sexes in response to the rapamycin treatment. The two-week rapamycin treatment had no effect on the serum clearance for ^125^I-insulin in males or females (Figure 7A,B). There was no effect of rapamycin treatment on the vascular marker, ^99m^Tc-albumin (data not shown), suggestive of an intact BBB. Additionally, there was no significant effect on ^125^I-insulin BBB transport (*p* = 0.055) nor the amount of ^125^I-insulin vascular binding due to the chronic rapamycin treatment (Figure 7C,D, Table 2). For females, the linear regression was not statistically significant, but the rates are similar to those observed in the past for ^125^I-insulin BBB transport [14]. 

**Figure 8:**
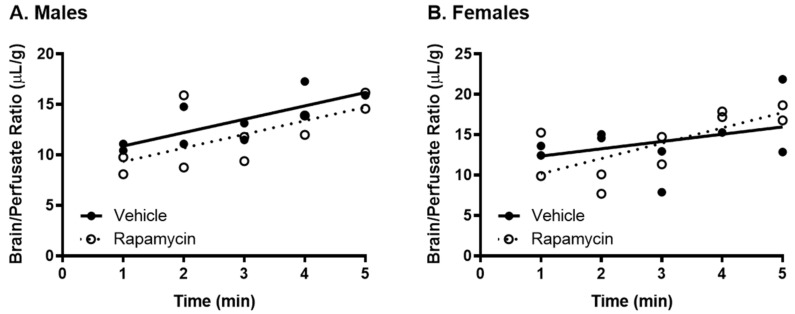
Effect of chronic rapamycin treatment on insulin BBB transport following transcardiac perfusion. The BBB pharmacokinetics of ^125^I-insulin in the absence of serum factors was unaltered by rapamycin in both (**A**) males and (**B**) females. Pharmacokinetic data is represented in Table 3.

Since serum factors are known to affect insulin BBB transport and can mask BBB transport changes and since rapamycin has been shown to impact the levels of serum factors known to affect insulin transport, we wanted to investigate BBB transport in the absence of serum factors. There was no impact of rapamycin on the basal ^125^I-insulin BBB pharmacokinetics in the absence of serum factors (Figure 8, Table 3), suggesting chronic rapamycin treatment does not have a direct effect on the BBB insulin transporter expression or activity. 

### 3.5. Effect of Chronic Rapamycin Treatment on Serum Triglyceride Level

**Figure 9:**
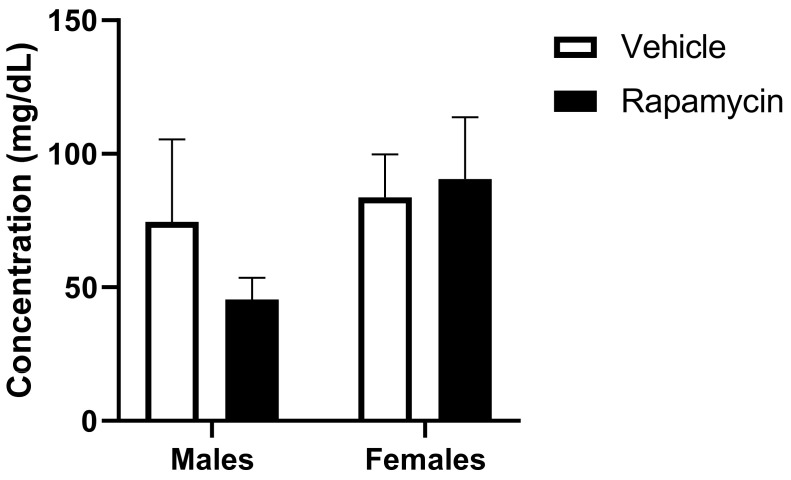
Serum triglyceride levels following rapamycin treatment. There were no significant changes in the serum triglyceride concentrations due to the rapamycin treatment or sex; *n* = 4/group.

As previous studies have shown serum triglyceride levels can impact insulin BBB transport [4], we determined whether there were changes in the basal serum triglyceride levels due to a two-week rapamycin treatment. The dose of rapamycin delivered did not elicit a significant change in serum triglyceride levels (Figure 9).

## 4. Discussion

We investigated the effects of rapamycin treatment on the basal levels of insulin vascular binding, receptor signaling, and BBB pharmacokinetics in male and female mice. While we found an acute spike in the blood rapamycin levels had no impact on insulin BBB transport, it did decrease vascular binding in male mice. We also found a short-term chronic, daily treatment with rapamycin led to alterations in insulin signaling in endothelial cells. Chronic rapamycin differentially affected body weight gain, serum glucose, and the serum levels of other metabolic hormones and cytokine levels due to sex. There was no significant effect on the BBB insulin pharmacokinetics due to chronic rapamycin treatment in the presence or absence of serum factors. There were also no changes in the serum triglyceride levels following the rapamycin treatment paradigm. 

An acute increase in the blood rapamycin levels led to a small, but significant decrease in insulin vascular binding in the whole brain. A decrease in binding could lead to changes in the intracellular insulin receptor signaling, which is known to affect expression the levels of various proteins, including cell adhesion molecules [8]. Changes in these alternative proteins within the brain endothelial cells could affect signals relayed to the CNS. We observed changes in insulin receptor signaling following chronic rapamycin treatment in a predominantly endothelial cell tissue, the aorta. Aortic and brain endothelial cells are regulated differentially but the aorta can be a predictor of what is occurring in the brain endothelial cells. While not quite reaching statistical significance using a two-way ANOVA, there was a trend in decreased pAkt Ser^473^ protein levels compared to the total Akt in males in the aorta. This protein difference in males due to the rapamycin treatment could be due to the decreased body weight gain present in males treated with rapamycin compared to the vehicle controls. There was a significant increase in the MAPK protein levels relative to β-actin due to the rapamycin treatment as well as in the females compared to the males, suggesting a dysregulation of this signaling molecule. The inhibition of mTOR using rapamycin has been shown to have an impact on the MAPK pathway [15,16]. These studies suggest rapamycin has a sexual dimorphic effect on MAPK signaling in endothelial cells, with a greater effect elicited in females.

While others have reported a decrease in body weight in male mice with rapamycin treatment [6,9], a decrease in body weight for C57BL6/J female mice has also been reported [9]. We observed a decrease in body weight only in males. The lack of difference in our study between sexes could be due to the differences in mouse strains (CD-1 vs. C57BL6/J) or the rapamycin treatment length. Similar to our findings, rapamycin has previously been shown to impair glucose tolerance following 2 weeks of daily injections of a fourfold lower concentration of rapamycin than the one used in the current study [17]. Five weeks of daily rapamycin treatment also led to an increase in the serum glucose levels of mice fasted overnight [17]. While we did not observe a significant effect of rapamycin on the fasted serum insulin levels, we did observe differences in the brain insulin levels in females only. Although this could be due to changes in insulin BBB transport, it is more likely due to changes in insulin brain degradation, based on our results below investigating BBB transport. An increase in the brain/serum ratio for insulin in the vehicle females was unexpected. Sex differences in the insulin brain/serum ratios have not been investigated. However, it is known women are at an increased risk for Alzheimer’s disease, which could be linked to alterations in CNS insulin signaling and thus differences in the brain/serum insulin ratios. The serum resistin levels were significantly decreased due to the rapamycin treatment, independent of sex. Acute caloric restriction, which is thought to act similar to rapamycin treatment, decreases resistin levels [18]. The serum GLP-1 levels were significantly increased due to the rapamycin treatment which is similar to what occurs with fasting [19]. PAI-1 is often considered a marker of insulin resistance. However, it is interesting that we observed an interaction due to sex and the rapamycin treatment on the serum levels of PAI-1. The serum insulin and glucose levels were higher in the males which could account for the differences observed in males versus females for the PAI-1 levels.

As rapamycin is used in the clinic as an immunosuppressant and since cytokines can affect insulin BBB transport, we wanted to measure the serum cytokine levels following our rapamycin treatment paradigm. We found the rapamycin treatment was able to increase the IL-2, IL-3, and IL-17 levels. These cytokines are all produced by T cells. IL-2 is known to impact the Akt/mTOR and MAPK/ERK pathways and works in conjunction with IL-3. IL-17 is a pro-inflammatory cytokine that is known to aid in the release of chemokines, recruiting immune cells and working in conjunction with TNF-α, for which there was a trend towards increased levels due to the rapamycin treatment. However, the degree of change for these cytokines was unable to alter insulin BBB transport, as discussed below, suggesting a greater level of cytokine production is needed or a different combination of cytokine increases is necessary to alter insulin BBB transport.

There was no difference in the transport rate of insulin across the BBB following two weeks of rapamycin treatment. In the males, there was a trend in a decreased transport rate due to rapamycin (*p* = 0.055). However, there was no effect in females. Additionally, the level of insulin vascular binding was similar between the two treatment groups. The lack of differences in insulin vascular binding here compared to the acute administration of rapamycin could be due to a couple different factors. First, the brain endothelial cells could have adjusted to the chronic exposure of rapamycin administration and therefore did not have a strong impact on vascular binding. Second, acute administration of rapamycin could lead to differences in the levels of serum factors that could also impact insulin binding. The trending difference in males could be due to the metabolic changes observed in the male mice due to the rapamycin treatment. Various physiological factors are known to alter insulin BBB transport [20]. However, it was anticipated that rapamycin would increase BBB transport rather than decrease transport based on previous studies showing similarities between the beneficial effects of rapamycin and insulin on cognition. While changes in BBB transport can be masked by serum factors, there was still no effect of rapamycin treatment on insulin BBB transport in the absence of serum factors. 

Triglycerides were one mechanism we hypothesized rapamycin could impact insulin BBB transport. However, our rapamycin treatment regimen did not alter the serum triglyceride levels which could be one reason we did not observe significant differences in insulin BBB transport. Previous reports in growth hormone receptor knockout male mice showed that a 4 mg/kg rapamycin treatment administered every other day for 20 weeks was able to increase the serum triglyceride levels by approximately 12% [6]. A study in humans reported an increase in the serum triglyceride levels by approximately 47% following a 10 mg/day dose of sirolimus for 42 days as tolerated [5]. It should be noted this patient population was chosen for this longer treatment period due to an initial hyperlipidemic response to a short-term treatment of sirolimus. Lastly, in one final study looking at the serum triglyceride levels in mice fed rapamycin orally, no differences were observed [21]. These studies suggest the genetic background and propensity to respond to rapamycin can alter the serum triglyceride levels differently.

Our findings suggest that rapamycin can alter the metabolic factors and insulin receptor signaling and binding on endothelial cells in a sexually dimorphic way. However, chronic rapamycin treatment is unable to alter insulin BBB transport. The differences in metabolism between males and females should be taken into consideration in planning future studies. Longer treatments (13+ weeks) with rapamycin have been shown to improve cognition [22]. Since evidence for testing rapamycin treatment in humans with Alzheimer’s disease is becoming greater [2], mechanisms by which rapamycin could improve cognition in pre-clinical models should continue to be investigated. Therefore, investigating the impact of long-term rapamycin treatment on insulin BBB transport, especially in mouse models of Alzheimer’s disease, still warrants further investigation as a mechanism for contributing to the cognitive improvements due to rapamycin.

## Figures and Tables

**Table 1:** Insulin BBB pharmacokinetics following an acute rapamycin i.v. injection based on Figure 1.

Treatment	K_i_ (µL/g∙min)	K_i_ *p*-Value	r	V_i_ (µL/g)	V_i_ *p*-Value
Vehicle	0.672 ± 0.15	0.63	0.89	11.1 ± 1.6	0.02
Rapamycin	0.541 ± 0.13		0.83	10.1 ± 1.3	

**Table 2:** Insulin BBB pharmacokinetics following chronic rapamycin treatment based on Figure 7.

Sex	Treatment	K_i_ (µL/g∙min)	K_i_ *p*-Value	r	V_i_ (µL/g)	V_i_ *p*-Value
Male	Vehicle	1.85 ± 0.13	0.055	0.99	2.1 ± 0.7	0.452
	Rapamycin	1.32 ± 0.19		0.93	4.0 ± 1.0	
Female	Vehicle	0.77 ± 0.45 (ns)	0.945	0.64	10.7 ± 4.4	0.721
	Rapamycin	0.73 ± 0.34 (ns)		0.72	9.9 ± 2.9	

**Table 3:** Insulin BBB pharmacokinetics in the absence of serum factors following chronic rapamycin treatment based on Figure 8.

Sex	Treatment	K_i_ (µL/g∙min)	K_i_ *p*-Value	r	V_i_ (µL/g)	V_i_ *p*-Value
Male	Vehicle	1.32 ± 0.42	0.97	0.766	9.6 ± 1.3	0.122
	Rapamycin	1.35 ± 0.53		0.672	8.0 ± 1.7	
Female	Vehicle	0.90 ± 0.83 (ns)	0.35	0.378	11.5 ± 2.7	0.860
	Rapamycin	1.89 ± 0.63		0.726	8.3 ± 2.1	

## Supplementary Materials

The following are available online at https://www.mdpi.com/article/10.3390/medsci9030056/s1, Figure S1. Western blots for Aorta (Figure 5).

## References

[1] Johnson S.C., Sangesland M., Kaeberlein M., Rabinovitch P.S. (2015). Modulating mTOR in aging and health. Interdiscip. Top. Gerontol..

[2] Kaeberlein M., Galvan V. (2019). Rapamycin and Alzheimer’s disease: Time for a clinical trial?. Sci. Transl. Med..

[3] Kennedy B.K., Lamming D.W. (2016). The Mechanistic Target of Rapamycin: The Grand ConducTOR of Metabolism and Aging. Cell Metab..

[4] Urayama A., Banks W.A. (2008). Starvation and triglycerides reverse the obesity-induced impairment of insulin transport at the blood-brain barrier. Endocrinology.

[5] Morrisett J.D., Abdel-Fattah G., Hoogeveen R., Mitchell E., Ballantyne C.M., Pownall H.J., Opekun A.R., Jaffe J.S., Oppermann S., Kahan B.D. (2002). Effects of sirolimus on plasma lipids, lipoprotein levels, and fatty acid metabolism in renal transplant patients. J. Lipid Res..

[6] Fang Y., Hill C.M., Darcy J., Reyes-Ordonez A., Arauz E., McFadden S., Zhang C., Osland J., Gao J., Zhang T. (2018). Effects of rapamycin on growth hormone receptor knockout mice. Proc. Natl. Acad. Sci. USA.

[7] Xaio H., Banks W.A., Niehoff M.L., Morley J.E. (2001). Effect of LPS on the permeability of the blood-brain barrier to insulin. Brain Res..

[8] Rask-Madsen C., Li Q., Freund B., Feather D., Abramov R., Wu I.H., Chen K., Yamamoto-Hiraoka J., Goldenbogen J., Sotiropoulos K.B. (2010). Loss of insulin signaling in vascular endothelial cells accelerates atherosclerosis in apolipoprotein E null mice. Cell Metab..

[9] Bitto A., Ito T.K., Pineda V.V., LeTexier N.J., Huang H.Z., Sutlief E., Tung H., Vizzini N., Chen B., Smith K. (2016). Transient rapamycin treatment can increase lifespan and healthspan in middle-aged mice. eLife.

[10] Lee N., Woodrum C.L., Nobil A.M., Rauktys A.E., Messina M.P., Dabora S.L. (2009). Rapamycin weekly maintenance dosing and the potential efficacy of combination sorafenib plus rapamycin but not atorvastatin or doxycycline in tuberous sclerosis preclinical models. BMC Pharmacol..

[11] Blasberg R.G., Fenstermacher J.D., Patlak C.S. (1983). Transport of alpha-aminoisobutyric acid across brain capillary and cellular membranes. J. Cereb. Blood Flow Metab..

[12] Patlak C.S., Blasberg R.G., Fenstermacher J.D. (1983). Graphical evaluation of blood-to-brain transfer constants from multiple-time uptake data. J. Cereb. Blood Flow Metab..

[13] Zar J.H. (1984). Biostatistical Analysis.

[14] Rhea E.M., Rask-Madsen C., Banks W.A. (2018). Insulin transport across the blood-brain barrier can occur independently of the insulin receptor. J. Physiol..

[15] Rastogi R., Jiang Z., Ahmad N., Rosati R., Liu Y., Beuret L., Monks R., Charron J., Birnbaum M.J., Samavati L. (2013). Rapamycin induces mitogen-activated protein (MAP) kinase phosphatase-1 (MKP-1) expression through activation of protein kinase B and mitogen-activated protein kinase kinase pathways. J. Biol. Chem..

[16] Carracedo A., Ma L., Teruya-Feldstein J., Rojo F., Salmena L., Alimonti A., Egia A., Sasaki A.T., Thomas G., Kozma S.C. (2008). Inhibition of mTORC1 leads to MAPK pathway activation through a PI3K-dependent feedback loop in human cancer. J. Clin. Investig..

[17] Arriola Apelo S.I., Neuman J.C., Baar E.L., Syed F.A., Cummings N.E., Brar H.K., Pumper C.P., Kimple M.E., Lamming D.W. (2016). Alternative rapamycin treatment regimens mitigate the impact of rapamycin on glucose homeostasis and the immune system. Aging Cell.

[18] Jakobsdottir S., van Nieuwpoort I.C., van Bunderen C.C., de Ruiter M.B., Twisk J.W., Deijen J.B., Veltman D.J., Drent M.L. (2016). Acute and short-term effects of caloric restriction on metabolic profile and brain activation in obese, postmenopausal women. Int. J. Obes..

[19] Yamaoka-Tojo M., Tojo T., Takahira N., Matsunaga A., Aoyama N., Masuda T., Izumi T. (2010). Elevated circulating levels of an incretin hormone, glucagon-like peptide-1, are associated with metabolic components in high-risk patients with cardiovascular disease. Cardiovasc. Diabetol..

[20] Rhea E.M., Banks W.A. (2019). Role of the Blood-Brain Barrier in Central Nervous System Insulin Resistance. Front. Neurosci..

[21] Den Hartigh L.J., Goodspeed L., Wang S.A., Kenerson H.L., Omer M., O’Brien K.D., Ladiges W., Yeung R., Subramanian S. (2018). Chronic oral rapamycin decreases adiposity, hepatic triglycerides and insulin resistance in male mice fed a diet high in sucrose and saturated fat. Exp. Physiol..

[22] Spilman P., Podlutskaya N., Hart M.J., Debnath J., Gorostiza O., Bredesen D., Richardson A., Strong R., Galvan V. (2010). Inhibition of mTOR by rapamycin abolishes cognitive deficits and reduces amyloid-beta levels in a mouse model of Alzheimer’s disease. PLoS ONE.

