# Supplementary material for: Effects of Rapamycin on Insulin Brain Endothelial Cell Binding and Blood–Brain Barrier Transport
Source: Med Sci (Basel). 2021 Aug 25;9(3):56. doi: 10.3390/medsci9030056 (PMC8395935; doi:10.3390/medsci9030056)
Supplement: Supplementary file 1 [file medsci-09-00056-s001.zip › medsci-1193836-supplementary.pdf]

medical sciences

MDPI

Article

Supplementary Materials:

Effects of Rapamycin on Insulin Brain Endothelial Cell Binding and Blood–Brain Barrier Transport

Steven Nguyen [1], William A. Banks [2,3] and Elizabeth M. Rhea [2,3,*]

[1] University of Washington, Seattle, WA 98195, USA.
[2] Department of Medicine, Division of Gerontology and Geriatric Medicine, University of Washington, Seattle, WA 98195, USA.
[3] Research and Development, Veterans Affairs Puget Sound Health Care System, Seattle, WA 98108, USA.
[*] Correspondence: meredime@uw.edu

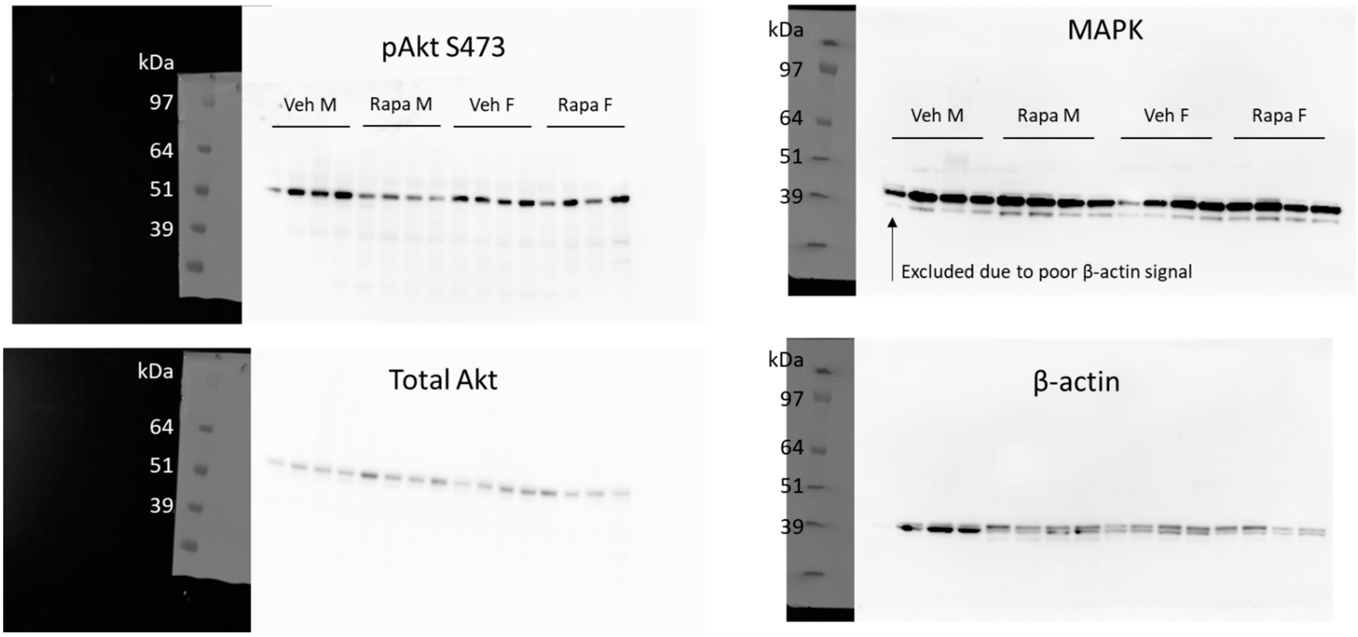

Figure S1. Western blots for Aorta (Figure 5).
